# Arbuscular Mycorrhiza Enhances Biomass Production and Salt Tolerance of Sweet Sorghum

**DOI:** 10.3390/microorganisms7090289

**Published:** 2019-08-23

**Authors:** Fayuan Wang, Yuhuan Sun, Zhaoyong Shi

**Affiliations:** 1 College of Environment and Safety Engineering, Qingdao University of Science and Technology, Qingdao 266042, China; 2 College of Agriculture, Henan University of Science and Technology, Luoyang 471023, China; 3 Key Laboratory of Soil Resources and Environment in Qianbei of Guizhou Province, Zunyi Normal University, Zunyi 563002, China

**Keywords:** sweet-sorghum, arbuscular mycorrhizal fungi, salt stress, bioenergy, saline soil

## Abstract

Arbuscular mycorrhizal (AM) fungi (AMF) are widely known to form a symbiosis with most higher plants and enhance plant adaptation to a series of environmental stresses. Sweet sorghum (*Sorghum bicolor* (L.) Moench) is considered a promising alternative feedstock for bioalcohol production because of its sugar-rich stalk and high biomass. However, little is known of AMF benefit for biomass production and salt tolerance of sweet sorghum. Here, we investigated the effects of *Acaulospora mellea* ZZ on growth and salt tolerance in two sweet sorghum cultivars (Liaotian5 and Yajin2) under different NaCl addition levels (0, 0.5, 1, 2, and 3 g NaCl/kg soil). Results showed AMF colonized the two cultivars well under all NaCl addition levels. NaCl addition increased mycorrhizal colonization rates in Yajin2, but the effects on Liaotian5 ranged from stimulatory at 0.5 and 1 g/kg to insignificant at 2 g/kg, and even inhibitory at 3 g/kg. High NaCl addition levels produced negative effects on both AM and non-AM plants, leading to lower biomass production, poorer mineral nutrition (N, P, K), higher Na^+^ uptake, and lower soluble sugar content in leaves. Compared with non-AM plants, AM plants of both cultivars had improved plant biomass and mineral uptake, as well as higher K^+^/Na^+^ ratio, but only Yajin2 plants had a low shoot/root Na ratio. AM inoculation increased the activities of superoxide dismutase (SOD), peroxidase (POD), and catalase (CAT), and soluble sugar content in leaves. Overall, both cultivars benefited from mycorrhization, and Yajin2 with less salt tolerance showed higher mycorrhizal response. In conclusion, AMF could help to alleviate the negative effects caused by salinity, and thus showed potential in biomass production of sweet sorghum in saline soil.

## 1. Introduction

Soil salinization is among the most common environmental problems. It is estimated approximately 7% of the Earth’s land surface area is salt-affected [1]. Excess ions, such as Na^+^ and Cl^−^, in saline soil generally depress plant growth and development via osmotic stress, ionic toxicity, nutritional imbalance, and oxidative damage [2]. All the physiological and biochemical processes involved in plant growth and development, such as photosynthesis, protein synthesis, and energy and lipid metabolism, are negatively impacted by salt stress [3]. Serious salt stress can lead to decreased plant productivity and even death of the whole plant [3].

Sweet sorghum is one of the sugar-rich crops with great potential for biofuel ethanol production because it produces high biomass containing both soluble (glucose and sucrose) and insoluble (cellulose and hemicellulose) carbohydrates [4]. The crop is also known as the “camel among crops” due to its wide adaptability and high resistance to abiotic stresses, including drought, salinity, waterlogging, and heavy metals [4]. Therefore, it is highly recommended to be grown on marginal lands with natural and/or artificial disadvantages [5]. In China, because of a large population and a low per capita arable land, the production of energy plants on marginal lands is recommended to ensure the security of both energy and food supply [5]. It’s of great significance to study plant performance of sweet sorghum on marginal lands.

Soil beneficial microorganisms are known to stimulate plant growth and enhance plant resistance to salt stress [6,7]. Among them, arbuscular mycorrhizal (AM) fungi (AMF) can form a mutualistic symbiosis with the majority of vascular plants in terrestrial environments and play significant roles in improving mineral nutrition and resistance of host plants [8,9]. AMF can promote plant growth and crop production under saline conditions [1,10,11]. The AM fungus isolated from coastal vegetation has been shown to promote sorghum growth and to efficiently suppress Na^+^ translocation into the shoot [12]. Putatively, AMF may be suitable for energy plant, such as sweet sorghum, production on marginal lands, such as salty soil.

Previous studies have shown AMF colonize sweet sorghum well [13] and increase plant growth and mineral nutrients of N and P [14], suggesting their potential in sweet sorghum production. However, to our knowledge, little is known of whether AMF can contribute to plant growth and salt tolerance of sweet sorghum under salt stress. Our hypothesis is inoculation with AMF can benefit plant growth and salt stress of sweet sorghum. The present study aimed to investigate the effects of inoculation with *Acaulospora mellea* on growth and salt tolerance in two sweet sorghum cultivars grown under different NaCl addition levels.

## 2. Materials and Methods

### 2.1. Soil, AMF Inoculum, and Plants 

The soil was collected from the top layer of the agricultural experimental field at the Zhoushan campus, Henan University of Science and Technology. The sieved soil (2 mm) was autoclaved at 121 °C for 120 min to eliminate indigenous AMF and air-dried for 2 weeks. The soil was classified as fluvo-aquic soil, and soil texture was loamy, with a pH (soil/water ratio of 1:2.5, w/v) of 8.15, 2.98% organic matter, 0.93 g/kg total N, 0.46 g/kg total P, 11.81 g/kg total K, and 0.40 g/kg soluble salt. 

The AM fungus *Acaulospora mellea* ZZ, kindly provided by Institute of Soil Science, Chinese Academy of Sciences, was selected because it has been shown to promote plant growth and tolerance in copper-, and phoxim-polluted soil [15,16]. AM inoculum was propagated on maize grown in pots with sterilized sands as substrates. The sands containing AM spores (>50 spores/g) were harvested and air-dried for inoculation experiments.

Liaotian5 and Yajin2, two sweet sorghum [*Sorghum bicolor* (L.) Moench] cultivars widely grown in China, were used for the inoculation experiments. Seeds with uniform size were selected for plant culture experiment. 

### 2.2. Experimental Design and Procedure

The addition levels of NaCl were based on previous studies [17,18]. The experiment is a bifactorial design consisting of (1) five NaCl addition levels (0, 0.5, 1, 2, and 3 g NaCl/kg air-dried soil, representing different salinity levels); (2) two AM inoculation treatments (inoculation with or without *Acaulospora mellea* ZZ). After mixing with NaCl and air-drying, 2 kg soil was filled into each plastic pot with 17 cm height × 19 cm top diameter × 14 cm bottom diameter. For AM inoculation treatments, 100 g AM inoculum per pot was mixed thoroughly with the soil. For the non-inoculation treatments, 50 mL of AM inoculum filtrate was added to the soil after mixing with 100 g sterilized AM inoculum per pot, to provide similar non-AMF microbial communities [19]. Four replicates of each treatment were conducted, giving a total of 80 pots. 

Seeds of two cultivars were germinated at 28 °C in darkness (for about 48 h) after surface-sterilizing using 0.5% NaClO solution. Ten uniform seeds of each cultivar were sown in each pot. The pots were placed in a greenhouse with a light intensity of about 500–850 μmol/m^2^/s, the temperature of 25–36/15–25 °C (day/night), and relative humidity of 30–60%/65–90% (day/night). The plants were watered with tap water to maintain soil moisture of approximately 70% of field water holding capacity. 

### 2.3. Plant and Soil Analysis

Plants were harvested two months after sowing. Shoots and roots were separately collected and cleaned, and the fresh weights (FWs) were measured. Fresh subsamples of roots and leaves were used to assess root colonization rate and the activities of antioxidant enzymes, respectively. The remaining plant materials were oven-dried at 70 °C for 48 h, and then dry weights (DWs) were weighed. 

Root colonization rate was determined using an ink staining method proposed by Vierheilig et al. [20]. Briefly, fresh root samples were stained for 3 min in a boiling ink (5%)-vinegar solution after clearing in boiling 10% KOH solution for 3 min and then used for observation under higher magnification. The dried plant materials were ground and wet-digested using a mixture of concentrated HNO_3_ and HClO_4_ (4:1, v/v). Standard plant reference material (GBW07603, GSV-2), purchased from the Institute of Geophysical and Geochemical Exploration, Chinese Academy of Geological Sciences (Langfang, China), was analyzed for quality control. To determine the concentrations of K and Na in the digestion solution, inductively coupled plasma-atomic emission spectrometry (ICP-AES) (Varian AA240, Varian, Inc., Palo Alto, CA, USA) was used. P concentration was measured using Vanadium-molybdenum yellow colorimetry. After digesting with an H_2_SO_4_ and H_2_O_2_ (5:2, v/v) mixture, plant N concentration was estimated using the Kjeldahl method [21]. 

The activities of three antioxidant enzymes were estimated. Fresh leaves (0.5 g) were homogenized in liquid nitrogen in 5 mL 50 mM phosphate buffer (pH 7), containing 4% (w/v) polyvinylpolypyrrolidone (PVPP), 0.3% (v/v) Triton X-100, and 0.1 mM ethylenediaminetetraacetic acid (EDTA). The homogenate was centrifuged at 15,000 g for 20 min at 4 °C. The supernatant was used to assay the activities of enzymes, superoxide dismutase (SOD, EC 1.15.1.1), peroxidase (POD, EC 1.11.1.7), and catalase (CAT, EC 1.11.1.6). 

The SOD activity was estimated using the method modified by Giannopolitis and Ries [22]. Absorbance was recorded at 560 nm. One unit of enzyme activity (U) was defined as the quantity of SOD required to produce a 50% inhibition of the reduction of nitroblue tetrazolium (NBT). POD activity was based on the determination of guaiacol oxidation at 470 nm by H_2_O_2_ [23]. The CAT activity was assayed spectrophotometrically, according to Aebi [24]. One unit of CAT activity is defined as 1 mol of H_2_O_2_ consumed by the enzyme per min and is measured at 240 nm. 

Soluble sugar was determined using the anthrone method [25]. Briefly, 1 mL extract, of 0.1 g ground dried plant tissues, was added to 5 mL anthrone reagents and heated at 100 °C for 10 min. The absorbance was recorded at 625 nm using a calibration curve with D-glucose as a standard. 

### 2.4. Data Analysis 

The mycorrhizal response (MR) was calculated using the DWs of non-AM and AM plants [26].

(1) $$\mathrm{MR}\;=\;\frac{\mathrm{DWs}\;\mathrm{of}\;\mathrm{AM}\;\mathrm{plants}-\mathrm{DWs}\;\mathrm{of}\;\mathrm{non-AM}\;\mathrm{plants}}{\mathrm{DWs}\;\mathrm{of}\;\mathrm{non-AM}\;\mathrm{plants}}\;\times 100$$

Data analysis was performed using SPSS 22. Multiple comparisons using Tukey’s test following one-way ANOVA (*p* < 0.05) were performed to assess statistical significance between the different treatments. Two-way ANOVA was used to assess the significance of interactions among the factors NaCl addition levels and AM inoculation. 

## 3. Results and Discussion

### 3.1. Root Colonization Rate

**Figure 1:**
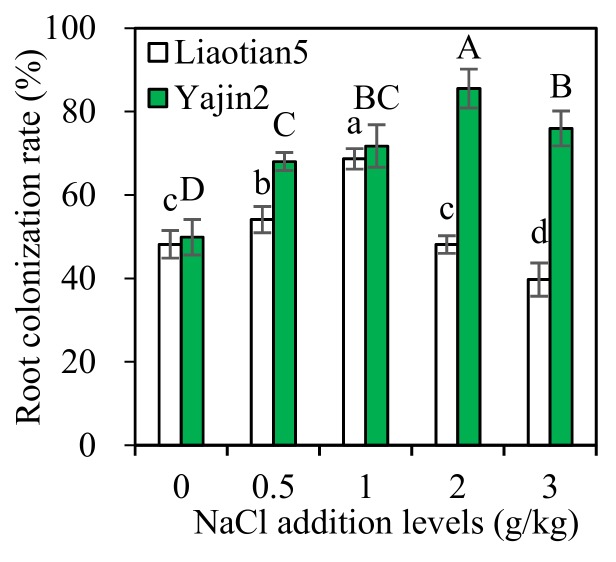
Root colonization rate (means ± SD, *n* = 4) of sweet sorghum under different NaCl addition levels. Different uppercase and lowercase letters above the bars indicate significant differences among the means of the same cultivar using a one-way ANOVA followed by the Tukey’s multiple range test (*p* < 0.05).

The plants of *Sorghum* are generally easily colonized by AMF and can be selected as optimal host plants for trap culture and propagation of AMF. Previous studies have shown sweet sorghum can form a symbiosis well with different AMF species [13,14], even under heavy metal-polluted conditions [27]. Our present results confirmed AMF successfully colonized the two sweet sorghum cultivars, irrespective of NaCl addition levels (Figure 1). However, the two cultivars responded variously to different NaCl addition levels. Compared to the control, root colonization rate of Liaotian5 was enhanced at 0.5 and 1 g/kg NaCl addition levels, not influenced significantly at 2 g/kg, but inhibited at 3 g/kg. Comparatively, the addition of NaCl simulated AM colonization in Yajin2, and the level 2 g/kg had the most pronounced effects. The variations in AM colonization in cultivars are probably due to host-AMF compatibility, which is cultivar dependent in various crops [28], such as maize, soybean, wheat, and barley. 

Generally, salt stress has detrimental effects on AM infectivity, spore germination, and hyphal growth [1,11,29]. Numerous studies have shown that the presence of NaCl reduced AM colonization and spore density, which are generally negatively correlated with salinity levels, soil electrical conductivity, and osmotic potential [1,30]. However, AMF have been known to occur widely in saline environments [1], indicating AMF can develop tolerance to salinity. Previous findings by Aliasgharzadeh et al. [31] showed that a relatively high AMF spore number (mean of 100 per 10 g soil) occurred in the severely saline soils of the Tabriz plains, and spore number remained unchanged with soil salinity. Root colonization rates were not decreased in coastal vegetation, receiving a high salinity of 200 mM [12]. Our present results confirmed slight and moderate salinity might stimulate AMF colonization, while the effects of severe salinity (3 g/kg) vary with the genotype of sweet sorghum.

### 3.2. Plant Biomass and Mycorrhizal Response (MR)

Overall, shoot and root dry weights (DWs) of both cultivars were remarkably influenced by AM inoculation, NaCl addition level, and the interactions between them (Table 1). As shown from the comparison of *F* values, AM inoculation displayed larger statistical significance on both shoot and root DWs than NaCl addition, and Yajin2 responded more significantly to AM inoculation than Liaotian5 did. 

**Figure 2:**
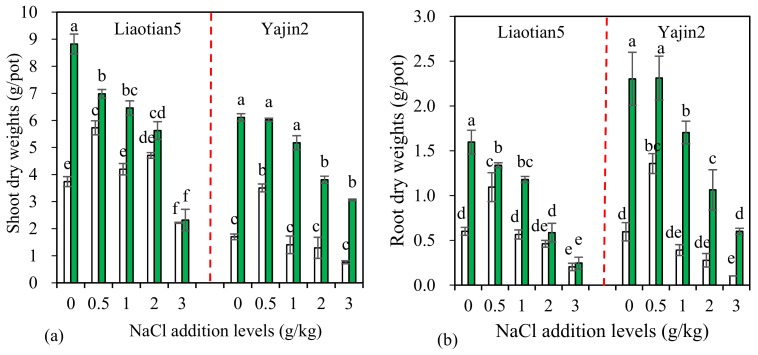
Shoot (**a**) and root (**b**) dry weights (means ± SD, *n* = 4) of sweet sorghum under different NaCl addition levels. The white and green columns represent non-AM inoculation and inoculation with *Acaulospora mellea* ZZ, respectively. Different letters above the bars indicate significant differences among different treatments of the same cultivar using a one-way ANOVA followed by the Tukey’s multiple range test (*p* < 0.05). Two-way ANOVA results are shown in Table 1.

One-way ANOVA results showed, compared with the zero addition treatments, shoot and root DWs of non-inoculated plants increased at 0.5 g/kg NaCl addition level, but decreased at 3 g/kg, and remained unchanged at 1 and 2 g/kg (Figure 2). Salt stress generally retards or decreases plant growth [3]. Previous studies have reported adverse impacts of salt stress on *Sorghum bicolor*, and varied responses to salt stress among cultivars [32,33], which are partially in accordance with our present results. The plants of Yajin2 receiving 3 g/kg NaCl suffered from severe chlorosis in leaves and nearly withered at harvest. When non-inoculated plants were exposed to 1–3 g/kg NaCl, compared to Yajin2, Liaotian5 had less growth reduction, indicating a stronger salt tolerance in this cultivar. 

Furthermore, beyond our expectation, exposure to 0.5–2 g/kg NaCl did not inhibit and, even, stimulate the growth of non-inoculated sweet sorghum (Figure 2). Previous studies have found low levels of NaCl can produce beneficial effects on the growth of sugar beet [34] and salt-resistant tomato cultivar Piazar [35], which are mainly due to an ability of plants to replace K^+^ by Na^+^. Particularly, Na is beneficial and even essential for some certain C_4_ plant species [36]. Thus, since sweet sorghum belongs to C_4_ plants, there is a possibility that this plant will benefit from an addition of NaCl.

Besides, different from ours, all the above-mentioned studies were conducted using sweet sorghum plants cultured in NaCl solution, where all Na^+^ and Cl^+^ ions are bioavailable and thus contribute to salt stress. However, under soil culture conditions, salt stress in plants may be alleviated by many other factors, such as organic matter [37] and microorganisms [1]. For example, sweet sorghum grown in fields with a soil salinity of 3.2 dS m^−1^ still produce sufficient juice, total sugar, and ethanol yields [38].

Another important finding is AM inoculation increased the growth of both cultivars treated with or without NaCl (Figure 2), with exceptions for Liaotian5 at 2 and 3 g/kg, confirming AMF have growth-promoting effects on sweet sorghum. The degree of enhancement varied with the NaCl addition levels. Even without significant effects on Liaotian5 at 2 and 3 g/kg NaCl addition level, AMF still showed a positive trend (Table 2). Salt stress can cause a reduction in chlorophyll content and depress plant growth by disrupting the photosynthetic process, but AMF often improve mineral nutrition and photosynthetic efficiency of plants, leading to a better growth [1,11]. Numerous studies have shown AMF confer salt tolerance to various plants and alleviate plant salt stress [1,10,11,29]. However, salt stress may inhibit the growth and colonization of AMF and the formation of effective symbiosis due to osmotic and/or toxic effects of salts [39]. Combining the results shown in Figure 1 and Figure 2, it can be found that plant growth-promoting effects of AMF correlated with AM colonization rate, and the lower colonization might account for their insignificant growth effects on Liaotian5 at 3 g/kg NaCl addition level.

There are different views on the relationship between AM colonization level and effects: one view is that the degree of colonization above 20% is regarded as effective [40], but another view is root colonization rate above 20% appears to be superfluous (but not harmful) [41]. In our present study, we have different findings: (1) the colonization rate at 3 g/kg NaCl reaching as high as 40% did not significantly increase the growth of Liaotian5; (2) the similar colonization rates in Liaotian5 at 0 and 2 g/kg NaCl addition level produced various growth effects; (3) both cultivars at 1 g/kg NaCl had similar colonization rates but displayed different mycorrhizal responses (Table 2). Altogether, the effectiveness of AMF and the minimal degree of colonization functioning effectively at least varied with salinity, plant species, and cultivars. 

MR reflects growth responsiveness of plants to AMF, which generally correlates with AMF species, plants, soil conditions, etc. [42]. Under salt stress, MR increases as salt concentrations increased [43], and varies with the isolates of fungus and species of the plant [18]. Our present study found MR varied with the cultivar and salinity: Yajin2 always had higher MR than Liaotian5, and the difference was particularly pronounced at 3 g/kg NaCl (Table 2). This can be attributed to the different salt tolerance in the two cultivars. Compared to Liaotian5, the cultivar Yajin2 was more sensitive to salinity, and, under salt stress, AM inoculation produced more significant effects. Our present results are consistent with the previous finding that under salt stress, the salt-sensitive tomato cultivar obtained more benefits from AM colonization than the salt-tolerant cultivar [30]. Thus, both salt tolerance and MR of cultivars should be considered for the use of AMF in the production of sweet sorghum in saline soils.

### 3.3. Plant Mineral Uptake and K^+^/Na^+^ Ratio

Two-way ANOVA results showed that shoot and root uptake, of N, P, K, and Na of both cultivars, was significantly influenced by AM inoculation, NaCl addition, and their interactions (Table 1). Comparing the *F* values, AM inoculation exhibited more significant effects on N, P, and K uptake in shoots and roots of Yajin2 than in Liaotian5, which suggests a higher mycorrhizal response in Yajin2 than in Liaotian5.

**Figure 3:**
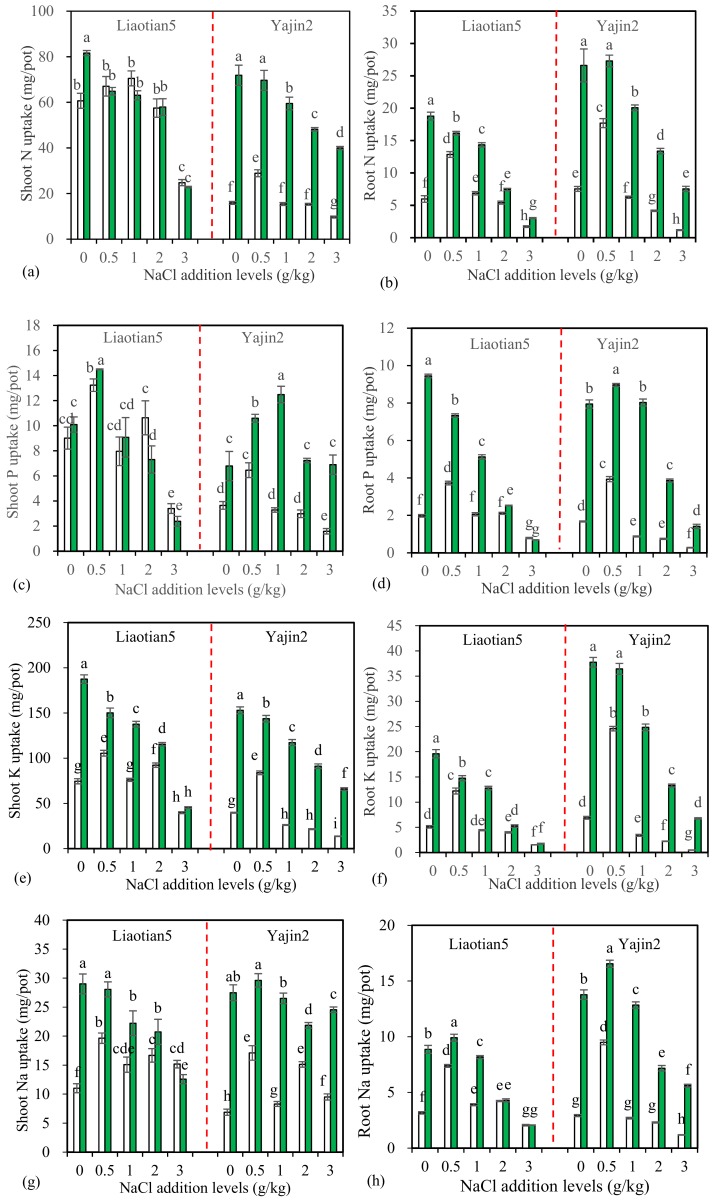
Uptake of N, P, K, and Na (means ± SD, *n* = 4) in shoots (**a**,**c**,**e**,**g**) and roots (**b**,**d**,**f**,**h**) of sweet sorghum under different NaCl addition levels. The white and green columns represent non-AM inoculation and inoculation with *Acaulospora mellea* ZZ, respectively. Different letters above the bars indicate significant differences among different treatments of the same cultivar using a one-way ANOVA followed by the Tukey’s multiple range test (*p* < 0.05). Two-way ANOVA results are shown in Table 1.

One-way ANOVA results showed NaCl addition affected N, P, and K uptake, but the results varied with addition level, cultivar, and AM inoculation (Figure 3a–h). In most cases, NaCl showed promoting effects on non-inoculated plants at low levels (such as 0.5 g/kg), but inhibitory effects at high levels especially at 3 g/kg. However, in comparison with the zero NaCl addition treatments, 0.5 g/kg NaCl decreased root uptake of N, P, and K in inoculated Liaotian5, but did not show inhibitory effects in inoculated Yajin2. Low levels of Na^+^ may function as beneficial nutrients and thus promote plant growth and nutrient uptake. However, high levels of Na^+^ not only inhibit the growth of roots and their activity to absorb nutrients but also compete with the uptake of other nutrients (especially K), resulting in nutrient deficiency (such as N, Ca, Mg, K, P, Fe, and Zn) [3]. Our present results support previous findings that salinity decreased plant mineral nutrition, but differences occurred between cultivars [30,35]. It is of significance to breed salt-tolerant cultivars suitable for production in saline soil via genetic engineering.

The most significant contribution of AMF for plants is a nutrient improvement (especially immobile soil nutrients, such as P) [9]. Under salt stress, AMF can increase plant uptake of nutrients, including P, N, K, Ca, Mg, and Zn [1]. In the current experiment, AM inoculation substantially increased the uptake of N, P, and K in both shoots and roots of Yajin2 at all NaCl addition levels, and the uptake of K in shoots, and of N, P, and K in roots of Liaotian5 at 0–2 g/kg NaCl (Figure 3a–f). AM effects varied with cultivars and NaCl addition levels. Yajin2 responded more significantly to AM inoculation than Liaotian5 (Table 1), which is in accordance with the results from plant biomass (Figure 2). However, AM inoculation did not significantly affect or, even, reduce the uptake of N, P, and K by Liaotian5 receiving 3 g/kg NaCl, which could explain why no significant AM benefits were observed for Liaotian5 at this salinity.

Maintaining a high K^+^/Na^+^ ratio is a key mechanism for plants counteracting Na^+^ stress, as K^+^ and Na^+^ balance bears importance for maintaining membrane potential and the activities of many cytosolic enzymes [2]. Plants in saline soil tend to take up more Na^+^, leading to decreased K^+^ uptake. Because of the competition between Na^+^ and K^+^ for binding sites essential for various cellular functions, low K^+^/Na^+^ ratio disrupts various metabolic processes [1,11]. Addition of NaCl increased Na concentrations in both cultivars, especially in roots (Table S1), and in most cases, decreased K^+^/Na^+^ ratios in shoots and roots (Figure S1), which might partly explain the salt toxicity in plants. Compared to Yajin2, Liaotian5 generally had lower shoot/root Na^+^ concentration ratio (Figure S2) and higher K^+^/Na^+^ ratio in shoots (Figure S1) at 2–3 g/kg NaCl addition levels, indicating this cultivar possesses a tolerance mechanism of preventing excess Na being translocated from roots to shoots. Furthermore, due to the deceased plant biomass by salt stress, Na uptake did not proportionally increase with the increasing NaCl addition levels (Figure 3g,h).

In addition to the K^+^/Na^+^ ratio, shoot/root Na^+^ ratio is also considered an indicator for the assessment of salt tolerance in AM plants [11]. AMF decrease Na^+^ concentrations in tomato shoots [44] and inhibit Na translocation from roots to shoots of sorghum [12]. Under saline conditions, AM colonization can increase K^+^ absorption, while decrease Na^+^ translocation to shoot tissues [1]. In our present study, the inoculated Yajin2 plants had lower shoot/root ratios for both Na concentrations and Na uptake than non-inoculated plants at 1–3 g/kg NaCl addition levels (Figure S2). AM inoculation enhanced plant biomass and/or K^+^ concentrations in them, leading to a higher K^+^ uptake (Figure 3e,f). Differently, the increased Na^+^ uptake by AM plants originated mainly from the increased biomass, and, in most cases, the Na^+^ concentrations decreased (Table S1). Thus, AM inoculation increased K^+^/Na^+^ ratios in both cultivars receiving 1–3 g/kg NaCl, but the effects were more pronounced in Yajin2 than in Liaotian5 (Figure S1). These findings confirm that (1) maintaining a high K^+^/Na^+^ ratio and a low shoot/root Na^+^ ratio is among the salt tolerance mechanisms employed by AM plants, and (2) this mechanism varies with the cultivars having different salt tolerance.

### 3.4. Plant Antioxidant Enzymes

**Figure 4:**
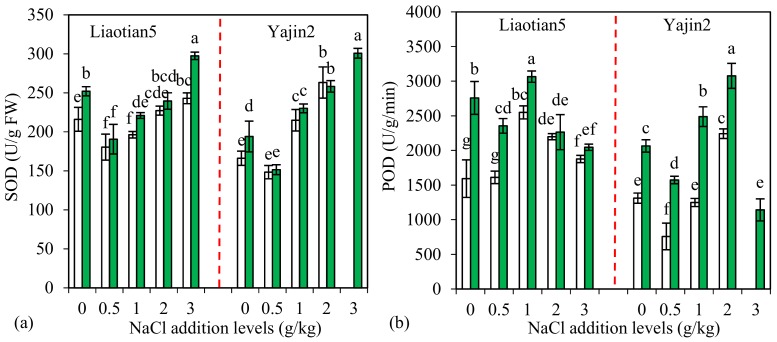

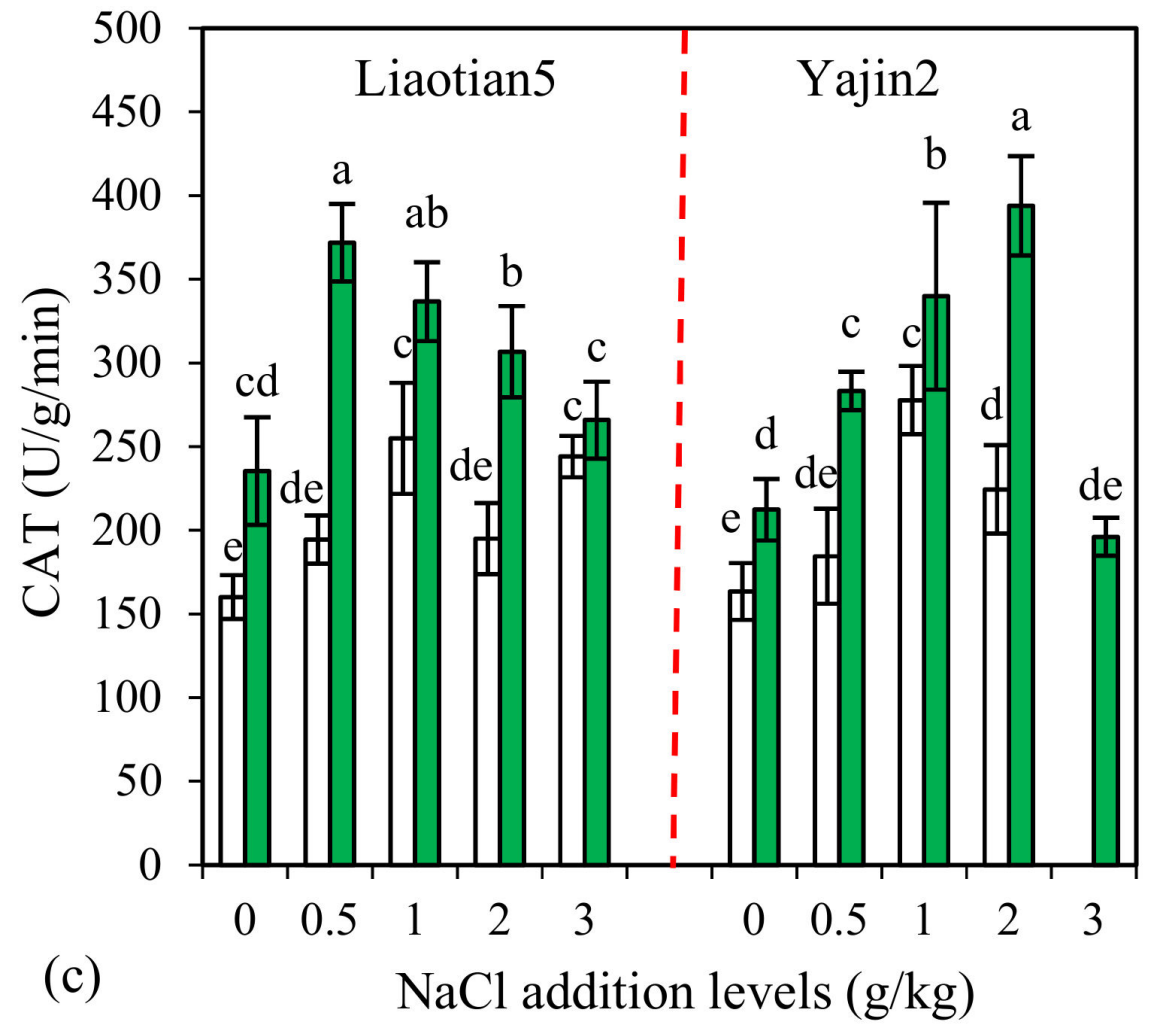
Activities (means ± SD, *n* = 4) of SOD (**a**), POD (**b**), and CAT (**c**) in leaves of sweet sorghum under different NaCl addition levels. The white and green columns represent non-AM inoculation and inoculation with *Acaulospora mellea* ZZ, respectively. Different letters above the bars indicate significant differences among different treatments of the same cultivar using a one-way ANOVA followed by the Tukey’s multiple range test (*p* < 0.05). Two-way ANOVA results are shown in Table 1. No fresh leaves of Yajin2 were sampled because of serious chlorosis at 3 g/kg NaCl addition level, and, thus, no enzyme activity was detected. SOD: superoxide dismutase; POD: peroxidase; CAT: catalase.

Reactive oxygen species (ROS) can react with functional materials, such as DNA, proteins, and lipids, and damage cell structure and function [1]. Salt stress may cause oxidative damages in plants by generating various ROS, such as singlet oxygen, O_2_^−^, OH^.^, OH^−^, and H_2_O_2_ [1,29]. SOD, POD, and CAT are among key antioxidant enzymes to scavenge ROS in plants. No fresh leaves of Yajin2 were sampled because of serious chlorosis at 3 g/kg NaCl addition level, and, thus, no enzyme activity was detected. Compared with the zero NaCl treatments, the activities of SOD, POD, and CAT in the two cultivars’ leaves (especially non-inoculated plants) were stimulated in several NaCl addition treatments (Figure 4a–c), which was in accordance with many other studies [3]. Maintaining a high level of antioxidants can eliminate excess ROS to avoid oxidative damages. However, different cultivars sometimes show various enzyme activity changes in response to NaCl addition. For example, POD activity was lower in Yajin2 than in Liaotian5 at 0.5 and 1 g/kg NaCl addition levels (Figure 4b).

One important biochemical mechanism employed by AM symbiosis to alleviate salinity stress is an increased activity of antioxidant enzymes, helping plants scavenge ROS and reduce oxidative damage to biomolecules [1,11]. AM plants generally have higher antioxidant enzyme activity than the non-mycorrhizal plants. Our present study also showed that in the vast majority of cases, AM plants exhibited increased activities of SOD, POD, and CAT (Figure 4a–c), but AM response varied with cultivars, enzyme species, and NaCl addition levels. AM effects were more significant on POD and CAT than SOD, and on Yajin2 than Liaotian5. AM colonization stimulates antioxidant enzyme activities in plants, but the response of the individual enzyme varies with host plant and AMF species [1]. The higher MR and lower salt tolerance of Yajin2 might account for its higher AM response to enzyme activities. Previous findings have shown higher activities of SOD and POD in mycorrhizal soybean plants than in nonmycorrhizal plants, but the activities of CAT and polyphenol peroxidase remain unchanged in both the plants [45]. SOD helps detoxify superoxide to H_2_O_2_, while the H_2_O_2_ generated will be detoxified by CAT and POD. ROS changes caused by salt stress may be varied in AM plants, and consequently, the ROS-scavenging enzymes may also respond differently to AM inoculation.

### 3.5. Soluble Sugar in Plants

Two-way ANOVA results showed that the content of soluble sugar in leaves of both cultivars was significantly influenced by AM inoculation and NaCl addition separately and their interactions (Table 1). Yajin2 responded more significantly to AM inoculation than Liaotian5.

**Figure 5:**
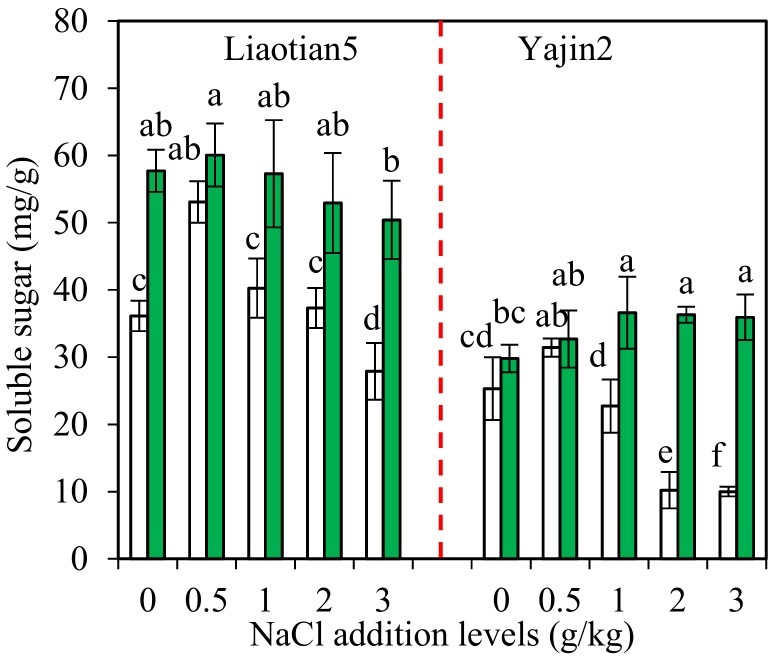
Soluble sugar content (means ± SD, *n* = 4) in leaves of sweet sorghum under different NaCl addition levels. The white and green columns represent non-AM inoculation and inoculation with *Acaulospora mellea* ZZ, respectively. Different letters above the bars indicate significant differences among different treatments of the same cultivar using a one-way ANOVA followed by the Tukey’s multiple range test (*p* < 0.05). Two-way ANOVA results are shown in Table 1.

Carbohydrates, such as sugars (glucose, fructose, sucrose, fructans) and starch, play important roles in osmoprotection, osmotic adjustment, and radical scavenging, in addition to carbon storage [3]. Plants can accumulate soluble sugars to adjust the osmotic potential and to maintain higher turgor pressure, which constitutes an important plant protection mechanism against osmotic stress (such as salinity and drought) [1]. Salt stress increases the contents of soluble sugars in various plants [3]. However, there are also different findings that soluble sugars are decreased or not changed by salinity [46,47]. In sweet sorghum, as salinity increases, the content of glucose and fructose decreases in one cultivar Keller while increases in another cultivar Sofra [48]. In our present study, NaCl addition exerted positive, negative, or insignificant effects on soluble sugar content in non-inoculated plants, depending on NaCl addition levels and cultivars (Figure 5). Low salinity tends to stimulate plants to accumulate soluble sugar, helping maintain a favorable osmotic potential. However, high salinity may cause damages in plants, leading to a poor ability to accumulate sugar. Furthermore, we also found Liaotian5 always had higher soluble sugar content than Yajin2, which can be explained by their different salt tolerance [48]. Combining with these results, our present results suggested sweet sorghum cultivars with different salt tolerance could adopt varied adjustment mechanisms in response to salt stress.

Salinity stress can produce osmotic stress and limit plants’ ability to take up water. AMF can adjust the osmotic potential of their host plants by increasing the concentration of organic products (e.g., proline, glycine betaine, carbohydrates, such as sucrose and mannitol), and thus improve the water use efficiency of plants [11]. As shown in Figure 5, soluble sugar content in leaves was significantly increased by AM inoculation at 1–3 g/kg NaCl addition levels, but remained unchanged at 0.5 g/kg. Numerous studies have found a positive correlation between sugar content and mycorrhization of the host plants under salinity stress, which is believed due to the sink effect of AMF demanding sugars from the shoot tissues and/or hydrolysis of starch to sugars in AM-colonized plants [1,11]. One possible reason is the increased photosynthetic efficiency and higher transport rate of carbon compounds from aerial parts to roots in AM plants. Since 0.5 g/kg of NaCl did not produce salinity stress, AM plants need not maintain a higher osmotic potential via changing sugar accumulation.

We also observed that AM inoculation induced greater increment in soluble sugar content in Yajin2 than in Liaotian5, especially at 3 g/kg NaCl addition level (Figure 5). The observations can be explained by the differences in their genotype-dependent salt tolerance and MR. Compared to Liaotian5, Yajin2 was more sensitive to salinity and grew worse under increased salinity; thus, it can benefit more from mycorrhization. Compared with the AM plants receiving no NaCl addition, AM plants receiving 3 g/kg NaCl had similar (in Liaotian5) or even higher soluble sugar content (in Yajin2), indicating adjustment of soluble sugar is an important salt tolerance mechanism in mycorrhizal plants.

Here, we provided nutritional and biochemical evidence that AMF alleviate salt-stress in different sweet sorghum cultivars. However, AMF protection mechanisms against salt stress may include nutritional, biochemical, physiological, and molecular responses [1]. For example, AMF can modulate the expression of genes involved in plant tolerance to salinity [11]. Glomalin-related soil protein (formerly known as glomalin) produced by AMF have a potential role in salt (Na^+^) immobilization [49]. Considering the importance of sweet sorghum for energy purposes, unknown mechanisms involved in salt tolerance of AM plants need more investigations using molecular and omics techniques, and advanced microscopy. In addition to enhanced soluble sugar content, AMF may also contribute to total sugar accumulation in sweet sorghum stalk, which will be of significance for its bioenergy purpose. It’s of great potential to select an effective cultivar-AMF combination for biomass production of sweet sorghum in saline soils.

## 4. Conclusions

In conclusion, NaCl addition affected the growth of sweet sorghum, but the effects varied with cultivars, NaCl addition levels, and AM colonization. High NaCl addition levels (3 g/kg) induced severe growth depression, poorer nutrition, and oxidative and osmotic stresses. Liaotian5 showed higher salt tolerance than Yajin2. Root colonization in Yajin2 was increased by NaCl addition, while colonization in Liaotian5 was reduced by 3 g/kg NaCl. AM plants showed higher salt tolerance than non-AM plants. AMF protected plants against salt stress via improving mineral nutrition, maintaining a high K^+^/Na^+^ ratio and a low shoot/root Na^+^ ratio, increasing antioxidant capacity by enhanced antioxidant enzymes and modifying osmotic adjustment by the accumulation of soluble sugar. Generally, both Yajin2 and Liaotian5 benefited from AM colonization under salinity stress, but Yajin2 showed higher mycorrhizal responses. Our results showed a potential of AMF in biomass production of sweet sorghum in saline soil.

## Figures and Tables

**Table 1:** Significance levels (*F* values) of AM (arbuscular mycorrhizal) inoculation, NaCl addition levels, and their interactions on measured variables based on two-way ANOVA results.

Cultivars	Variables	AM	NaCl	AM × NaCl
Liaotian5	Shoot dry weights	87.57 **	52.21 **	17.59 **
	Root dry weights	56.63 **	42.50 **	10.03 **
	Shoot N uptake	5.51 **	75.21 **	6.03 **
	Root N uptake	46.22 **	30.37 **	7.33 **
	Shoot P uptake	100.50 **	287.50 **	148.94 **
	Root P uptake	1239.95 **	528.92 **	271.13 **
	Shoot K uptake	127.99 **	11.93 **	6.02 **
	Root K uptake	139.20 **	142.84 **	63.41 **
	Shoot Na uptake	57.96 **	30.02 **	23.45 **
	Root Na uptake	99.32 **	602.32 **	104.72 **
	Soluble sugar	114.53 **	13.05 **	3.15 *
Yajin2	Shoot dry weights	205.268 **	21.297 **	3.619 **
	Root dry weights	141.251 **	35.910 **	5.646 **
	Shoot N uptake	645.61 **	25.96 **	12.47 **
	Root N uptake	119.94 **	35.08 **	4.21 *
	Shoot P uptake	640.37 **	56.23 **	26.02 **
	Root P uptake	1984.91 **	367.96 **	112.77 **
	Shoot K uptake	632.71 **	205.78 **	88.19 **
	Root K uptake	1503.20 **	940.96 **	247.30 **
	Shoot Na uptake	5.58 **	684.83 **	1284.87 **
	Root Na uptake	43.35 **	874.67 **	129.67 **
	Soluble sugar	160.91 **	8.93 **	20.12 **

Significance levels: * *p* < 0.05; ** *p* < 0.01.

**Table 2:** Mycorrhizal response (%) of sweet sorghum under different NaCl addition levels.

NaCl Addition Levels (g/kg)	Liaotian5		Yajin2	
	Shoots	Roots	Shoots	Roots
0	136	165.8	260	286.3
0.5	21.9	22.2	71.9	70.3
1	53.8	108.6	268.9	335.6
2	19.5	26.9	194.6	283.1
3	4.6	21.6	303.1	485.6

## Supplementary Materials

Supplementary materials can be found at https://www.mdpi.com/2076-2607/7/9/289/s1.

## Abbreviations

AM	arbuscular mycorrhizal
AMF	arbuscular mycorrhizal fungi
CAT	catalase
DWs	dry weights
FDs	fresh weights
MR	mycorrhizal response
POD	peroxidase
ROS	reactive oxygen species
SOD	superoxide dismutase
