# Supplementary material for: Arbuscular Mycorrhiza Enhances Biomass Production and Salt Tolerance of Sweet Sorghum
Source: Microorganisms. 2019 Aug 23;7(9):289. doi: 10.3390/microorganisms7090289 (PMC6780689; doi:10.3390/microorganisms7090289)
Supplement: Supplementary file 1 [file microorganisms-07-00289-s001.pdf]

Supplementary information

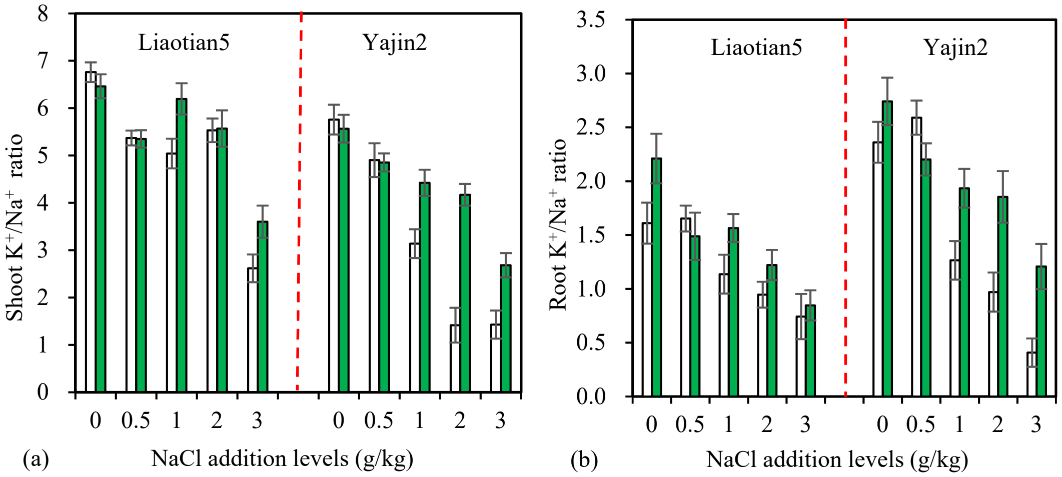

Figure S1. K^+/Na^+ ratio (mean values) in shoots (a) and roots (b) of sweet sorghum under different NaCl addition levels. The white and green columns represent non-AM inoculation and inoculation with *Acaulospora mellea* ZZ, respectively.

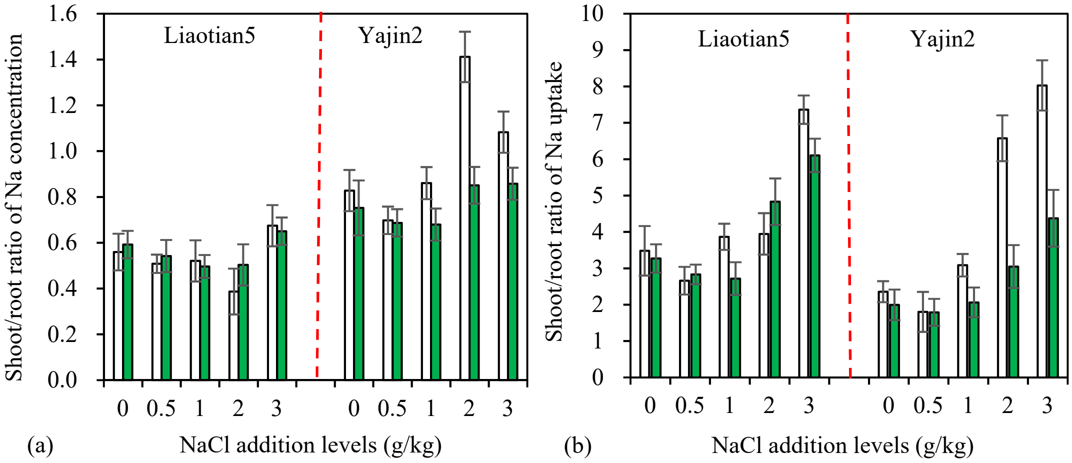

Figure S2. Shoot/root ratio of Na concentration (a) and uptake (b) of sweet sorghum under different NaCl addition levels. The white and green columns represent non-AM inoculation and inoculation with *Acaulospora mellea* ZZ, respectively.

Table S1. Concentrations of K and Na in shoots and roots of sweet sorghum under different NaCl addition levels

NaCl (g/kg)	Inoculation	Liaotian5				Yajin2			
		K concentration (g/kg)		Na concentration (g/kg)		K concentration (g/kg)		Na concentration (g/kg)	
		Shoots	Roots	Shoots	Roots	Shoots	Roots	Shoots	Roots
0	-M	19.92±0.76bc	8.48±0.42c	2.95±0.21f	5.27±0.18g	23.35±0.29bc	11.57±0.48f	4.05±0.31f	4.90±0.15c
	+M	21.24±0.54a	12.27±0.52a	3.29±0.19e	5.55±0.22f	25.04±0.61a	16.40±0.42b	4.50±0.22f	5.98±0.19f
0.5	-M	18.42±0.56d	7.84±0.21d	3.43±0.16de	6.75±0.11e	23.92±0.58b	18.11±0.33a	4.88±0.36e	6.99±0.16e
	+M	21.50±0.76a	11.03±0.39b	4.02±0.19c	7.41±0.23d	23.86±0.60b	15.76±0.47c	4.92±0.19e	7.16±0.14d
1	-M	18.11±0.41d	7.84±0.21d	3.59±0.31de	6.90±0.16e	18.59±0.31e	8.71±0.49g	5.92±0.30d	6.88±0.18e
	+M	21.31±0.49a	10.84±0.30b	3.44±0.33de	6.93±0.11e	22.66±0.65c	14.58±0.38d	5.12±0.18e	7.54±0.17e
2	-M	19.61±0.51c	8.66±0.30c	3.54±0.25de	9.16±0.11b	16.57±0.39g	8.04±0.19h	11.70±0.37b	8.29±0.18b
	+M	20.53±0.33ab	8.95±0.44c	3.69±0.38cd	7.32±0.24d	23.93±0.66b	12.50±0.27	5.74±0.13d	6.74±0.23d
3	-M	17.91±0.49d	7.54±0.13de	6.84±0.29a	10.14±0.32a	17.90±0.33f	4.71±0.28i	12.52±0.70a	11.56±0.23a
	+M	19.54±0.43c	7.06±0.34e	5.42±0.34b	8.34±0.09c	21.49±0.47d	11.28±0.23f	8.01±0.16c	9.34±0.21c

Note: -M and +M represent non-AM inoculation and inoculation with *Acaulospora mellea* ZZ, respectively. Different letters following the mean values (± SD) in the same column indicate significant differences among different treatments of the same cultivar using a one-way ANOVA followed by the Tukey's multiple range test ($p < 0.05$).
